# P-Glycoprotein Altered Expression in Alzheimer's Disease: Regional Anatomic Variability

**DOI:** 10.1155/2013/257953

**Published:** 2013-04-03

**Authors:** Brian Jeynes, John Provias

**Affiliations:** ^1^Department of Community Health Sciences, Faculty of Applied Health Sciences, Brock University, 500 Glenridge Avenue, St. Catharines, ON, Canada L2S 3A1; ^2^Department of Pathology & Molecular, Medicine [Neuropathology], Hamilton Health Sciences, McMaster University, Hamilton, 1280 Main Street West, Hamilton, ON, Canada L8S4L8

## Abstract

We investigated the expression of P-glycoprotein (P-gp) in brain samples of Alzheimer disease (AD) and normative brains (NM). Superior temporal cortex hippocampal and brainstem samples from 15 AD and NM brains were selected from comparable sites. P-gp positive capillaries and *β*-amyloid (A*β*) senile plaques (SP) were counted. Statistical analysis of the data was performed using nonparametric data analysis with Mann-Whitney, Kruskal-Wallis, and Spearman's tests. There were no significant differences in P-gp expression between superior temporal and hippocampus samples. However, there were significant differences in P-gp expression, when comparing brainstem with both hippocampal and superior temporal samples in both conditions (*P* < 0.012; *P* < 0.002 in NM cases and *P* < 0.001; <0.001 in AD cases); the brainstem has greater P-gp expression in each case and condition. In addition, there was a notable inverse negative correlation (*P* < 0.01) between P-gp expression and the presence of SPs in the AD condition superior temporal cortex. The results of this study suggest that there were significant site-dependent differences in the expression of P-gp. There may be an increased protective role for P-gp expression against amyloid deposition in the brainstem and in the superior temporal cortex of AD brains.

## 1. Introduction

Alzheimer's disease, which is largely a sporadic disease, increases in prevalence with age. In addition, in individual cases, the neuropathologic burden of Alzheimer disease, that is, neuronal neurofibrillary tangle and related dysfunction as well as beta amyloid (A*β*) senile plaques [SPs] increases progressively with time [1–3]. The hallmark lesions for AD are neurofibrillary tangles (NFTs) and senile plaques (SPs) [4]. A striking characteristic of these lesions is that they develop in particularly predictable areas of the brain and are completely or mostly absent in other areas [5, 6]. Neurofibrillary tangles, as they progressively populate neuronal populations of brain tissue, progress from the earliest lesion in the entorhinal cortex to involve neighbouring limbic cortex and then progressively through neocortex [7]. However, neurofibrillary tangles are typically and frequently found in key selected subcortical structures, chiefly, hippocampus, the nucleus basalis of Meynert, and the pontine locus ceruleus [8–11]. SPs develop as a result of a pathologic accumulation of A*β* proteins in the cerebral interstitium as well as within the walls of capillaries and larger cerebral vessels [12]. Senile plaques also have a characteristic distribution which is chiefly neocortical and limbic cortex [13]. Thus, the distribution of senile plaques does not entirely mirror that of neuronal neurofibrillary tangles. A*β* progressive deposition and accumulation in neural tissue, although less predictable than that of senile plaques, tends to follow a similar distribution, emphasizing the predominant pattern of limbic and neocortical deposition [6]. Whether there is a pathogenic relationship between neuronal neurofibrillary tangle development and senile plaque formation remains unresolved [14, 15]. An explanation for this remains elusive.

One factor that may explain this differential distribution is an area and site specific variation in the activity of one or more of the transendothelial transport proteins associated with the blood-brain barrier (BBB). The BBB is comprised of the endothelium and its accompanying basal lamina, pericytes, and astrocyte end-processes [16–18]. With few exceptions, the BBB is present throughout the brain. The “barrier” function of the BBB is maintained primarily by the tight junctions between endothelial cells and passive and active transendothelial transport mechanisms. Passive transport across the BBB of water, gases and electrolytes, and nonlipid-soluble materials occurs by diffusion. The transport of plasma proteins and nonlipid-soluble large organic requires active participation of intraendothelial transport mediators [19].

There have been a number of hypotheses relating to the overall etiology of Alzheimer's disease development, in particular to the accumulation of A*β* and senile plaque formation. The small percentage of cases which are familial is better understood in the relationship between the underlying genetic etiology and pathogenesis, which seems more directly linked to A*β* peptide abnormal processing and accumulation [20]. The majority of Alzheimer's disease cases, that is, sporadic nonfamilial forms, have a number of underlying pathogenic hypotheses. These include altered perfusion dynamics, perhaps as a result of atherosclerosis, altered capillary density, and the increase of cellular oxidative stress. However, currently, the most prominent hypothesis related to the accumulation of A*β* is the amyloid cascade hypothesis [21], though this hypothesis is challenged [22, 23]. This hypothesis presupposes the beta amyloid accumulation and deposition within the CNS as the primary driving process in AD disease evolution and is somehow linked to the development of neuronal dysfunction and perhaps the progressive tauopathy which develops.

That being the case, the question arises as to the mechanism leading to accumulation of A*β* within the CNS. Normally amyloid precursor protein and the A*β* peptide derived from this larger precursor protein moiety undergo continual processing and turnover moving both in and out of the brain parenchymal compartment, leading to a normal homeostatic condition [24]. If this homeostasis breaks down, the A*β* peptide may not be cleared from the brain interstitium, leading to its progressive accumulation to pathogenic levels. The regulation of A*β* transport and movement between the brain and nonbrain compartments occur at the blood-brain barrier [BBB] level. 

A number of BBB-related transport proteins have been demonstrated to be active in the homeostatic regulation of the transport of A*β* in and out of the brain. These include receptor for advanced glycation end products (RAGE), an A*β* influx facilitator and lipoprotein receptor-related protein (LRP) and P-glycoprotein (P-gp), and A*β* efflux facilitators [25, 26]. P-gp is expressed on the BBB luminal endothelial membrane, effectively expediting the release of A*β* into the vascular compartment. It cooperates with LRP, found on the abluminal endothelial membrane, to transport A*β*_40&42_ out of the brain and into the vascular compartment [27]. It is a critical component in the regulation of A*β* levels in the brain. Dysfunction of these transport protein systems then can potentially lead to a progressive and toxic accumulation of beta amyloid peptide within the CNS parenchymal compartment. Abnormal processing from the precursor protein, APP, to beta amyloid peptide through the activity of secretase enzyme systems, may also play a role in the rate of accumulation of beta amyloid [28]. Thus the interactions between factors regulating beta amyloid production (secretase systems) and the blood-brain barrier situated transport proteins which mediate net efflux under normal homeostasis appear to be the fundamental driving and regulatory systems controlling final levels of beta amyloid within the brain compartment.

Diminished overall vascular P-gp function has been shown to be associated with an increase in *β*-amyloid deposition [1, 2, 29, 30]. This decrement in function may be directly related to a decreased expression of endothelial-associated P-gp. 

In this study we investigated and compared the expression of capillary P-gp in different regions of the brain of normative and Alzheimer brains. The correlations of P-gp expression with NFTs and SPs in those regions were examined in order to determine whether there were site-dependant variations in P-gp activity resulting in the presence of NFTs and/or SPs. 

## 2. Methods

15 cases of sporadic Alzheimer's disease, as well as 15 cases of non-Alzheimer's, nondemented individuals dying of nonneurologic causes, were selected from the archival autopsy material of the HHS (Hamilton Health Sciences). Alzheimer's disease (AD) cases were assessed utilizing standard neuropathologic criteria including Braak and Braak staging [8] and CERAD probability indexing [31]. All 15 AD cases were free from any significant other degenerative confounding neuropathology and from significant intracranial vascular pathology or infarcts. Tissue blocks were paraffin processed. Sections were routinely stained with Luxol Fast Blue/hematoxylin and eosin. Routine diagnostic assessment included immunohistochemistry for tau (Dako, rabbit polyclonal, C-terminus specific), beta amyloid (Dako, mouse monoclonal n-terminus_8–17_), ubiquitin (Dako, rabbit polyclonal), and alpha-synuclein (Zymed, mouse monoclonal). Superior temporal and hippocampal cortical and brainstem blocks underwent further immunohistochemical assessment with antibodies to beta amyloid_1–40_ (ID Labs Inc., rabbit polyclonal), beta amyloid_1–42_ (ID Labs Inc., rabbit polyclonal), and P-gp (Abcam, Cambridge, MA, mouse monoclonal (C494)). All immunohistochemistry was performed on standard 7 µm tissue sections using Avidin Biotin Complex (ABC) methodology with hematoxylin nuclear counterstaining. AD cases were diagnosed utilizing standard criteria based on the presence of beta amyloid positive plaques and neuronal neurofibrillary tau degeneration. In all of the selected cases, areas of superior temporal (ST) and hippocampal (HC) cortices and brainstem (BS) were selected for quantitative analysis of neuronal neurofibrillary tangles beta amyloid plaques and for positive capillary P-gp expression. For each region, a start point was randomly determined and 10 contiguous fields were examined. All fields were examined at 200x magnification. In this manner, neurofibrillary tangles, senile plaques, and beta amyloid and P-gp positive capillaries were counted and total counts, as well as densities (counts per field area), were determined for each. Neurofibrillary tangles (NFTs) were counted when tau was seen within the neuronal cytoplasm in a juxtanuclear position to exclude tau positive dystrophic neurites and neuropil threads. All beta amyloid positive plaques (SPs) were counted, including diffuse, neuritic, and core forms. P-gp positive capillaries were counted, with capillaries being defined as vessels less than 10 µm in diameter and morphologically consisting of only an endothelium and basal lamina.

Statistical analysis of the data was performed using the nonparametric data Mann-Whitney, Rank Sum, and Kruskal-Wallis tests for comparison of Alzheimer and control group (normative) conditions and sites. The Spearman's nonparametric correlation test was used to correlate the different lesion data within a specific region and condition.

## 3. Results

Table 1 summarizes the case descriptions with respect to gender, age, and the neuropathologic data for each condition. Overall the gender distribution was equal. The mean age for the NM condition was 68.8 years (72.4, males; 65.3, females); and for the AD condition it was 78.8 (77.6, males; 79.9, females) years. The Braak and Braak stages and CERAD levels were consistent with the assigned conditions.

**Figure 1:**
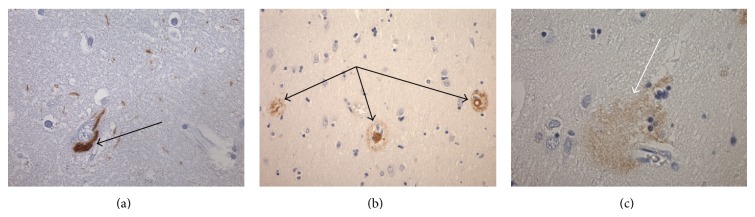
(a) Photomicrograph demonstrating a tau-stained NFT (×400). (b) Photomicrograph demonstrating *β*-amyloid bearing SPs (×200). (c) Photomicrograph demonstrating a diffuse *β*-amyloid SP lesion (×400). (All fields were superior temporal cortex samples in AD cases.)

Figures 1(a), 1(b), and 1(c) are representative photomicrographs demonstrating a tau-stained NFT (a) and a *β*-amyloid-stained SP (b) and diffuse SP (c) lesions. The tau and *β*-amyloid immunohistochemistry demonstrates the typical neurofibrillary tangle and range of plaque lesions observed and counted in this study.

As expected NFTs were most evident in AD cases in each site (*P* < 0.001); significant differences (*P* < 0.001) were observed when comparing sites, with ST samples having greater density than HC samples, which had greater density than BS in terms of lesion presence. 

**Figure 2:**
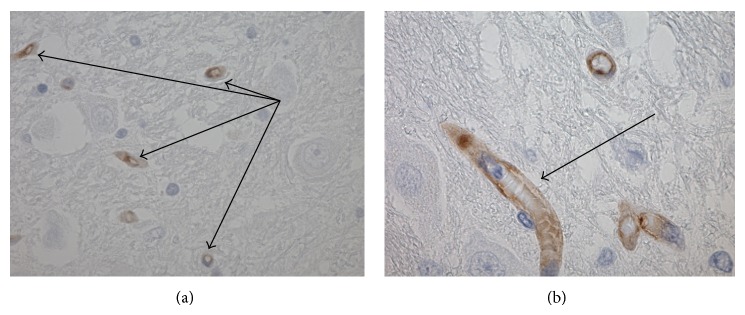
(a) Photomicrograph demonstrating P-gp positive capillaries (arrows) (AD sample from pons, ×400); (b) photomicrograph showing P-glycoprotein expression at the capillary endothelium (arrow) (AD sample from pons, ×600).

Figure 2(a) demonstrates P-gp labelled capillaries (arrows) illustrating an area of high P-glycoprotein expression in the pons. This is a region that has little or no *β*-amyloid accumulation and SPs. Capillary P-glycoprotein expression was located at the endothelium (Figure 2(b)). 

Table 2 presents the raw count data for the presence of SPs and P-gp positive capillaries in each condition and site.

With respect to SPs there was a significant difference in the presence of SPs when comparing NM and AD conditions (*P* at least <0.002). Further, when compared with the NM condition, there were more SPs in the AD condition. These AD condition differences were significantly different (*P* < 0.001). In both conditions brainstem samples had the lowest number of SP lesions. 

There were no statistically significant differences between the NM and AD conditions when comparing capillary P-gp expression in corresponding ST, HC, or BS regions. There appeared to be greater capillary P-gp expression in the Alzheimer condition in the HC and BS regions. In the ST region there appeared to be greater capillary expression for P-gp in the normative condition. 

There were no significant differences between superior temporal and hippocampus samples with respect to P-gp expression in either condition. However, there were significant differences in P-gp expression, when comparing brainstem with both hippocampal and superior temporal samples in both conditions (*P* < 0.012; *P* < 0.002 in NM cases and *P* < 0.001; <0.001 in AD cases), with the brainstem having greater P-gp expression in each case and condition (Table 3). 

The correlation analysis indicated that in the ST, only, there was a strong negative correlation between P-gp positive capillary density and senile plaque quantity (Table 4).

## 4. Discussion

This study has examined the expression of P-gp within the brainstem (pons), the hippocampus, and superior temporal cortex in both normative and Alzheimer disease brains. These sites where selected to encompass areas that typically have little (pons) or prominent (ST and hippocampus) AD pathology. The results show that P-gp expression is the highest within brainstem in relationship to the other two sites, that is, hippocampus and superior temporal cortex; the brainstem also has the lowest level of A*β* plaques. This finding of the highest level of P-gp expression within the brainstem is seen in both normative brains and Alzheimer condition brain tissue. When taking an A*β* rich region of the brain in Alzheimer's disease, such as the superior temporal cortex, significantly there was a negative correlation between A*β* senile plaque levels and P-gp expression. This inverse correlation was only seen within the superior temporal cortex; however, it should be considered that of the three anatomic sites examined, only this area of the brain showed significant A*β* burden and senile plaque levels as part of the Alzheimer disease condition. Other human and mouse model studies, as indicated earlier, have shown that a reduction in the global brain P-gp expression is associated with generalized increase in senile plaque development [29, 30, 32, 33]. 

It is important to consider that the distribution of the typical characteristic lesions of AD [5, 6], that is, neuronal tauopathy and A*β* senile plaque formation, remains unexplained. Certainly in relationship to beta amyloid senile plaques, the anatomic distribution may be a reflection of regional variations in the systems referred to which regulate A*β* levels, that is, secretase enzyme activity and the transport proteins. Whether or not neuronal dysfunction and tauopathy follow this distribution and anatomic pattern in a direct causal fashion or has some other more independent pathogeneses, remains as another open question beyond the consideration of this study. 

P-gp is one of the established coefflux transporters of beta amyloid [25]. Studies have shown P-gp levels to have reduced expression as a function of age [1–3]. Importantly, as indicated previously, some studies have shown that P-gp levels are inversely correlated with amyloid deposition in AD brains [29, 30, 32, 33]. It is possible, therefore, that there is a greater likelihood for increased pathogenesis for SP development as a function of aging. All brains were selected to be free of any significant non-Alzheimer-related neuropathology. Ideally, the NM group should be age-mated. Our NM group had a mean age of (68.8 years) and was younger than those in the AD population (78.8 years). This was so because of the availability of NM brains, particularly those that met our criteria. A younger comparative group would likely have less P-gp depletion than the AD group in this study and therefore have fewer SPs evident. However, we have a reason to believe that the inferences drawn from the results of this study remain valid in spite of this concern.

Some PET imaging studies have shown a regional difference in P-gp expression [34], including a study describing P-gp expression in human cerebellum and parahippocampal and middle temporal lobe regions [35]. Few studies have looked at variability and regional anatomic differences in P-glycoprotein expression within the human brain. In addition, no human studies have looked at P-gp expression in lesion prone and positive sites seen in Alzheimer's brain or in relationship to the development and accumulation of Alzheimer senile plaques and beta amyloid. Further investigation of P-gp expression within these regions of the AD brain would be key in order to establish variability in the local P-gp expression, which could play a role in the progressive beta amyloid accumulation and putative senile plaque pathogenesis. This is the first study looking at regional P-glycoprotein expression in human brains allowing for a comparison between normals and confirmed Alzheimer cases.

**Figure 3:**
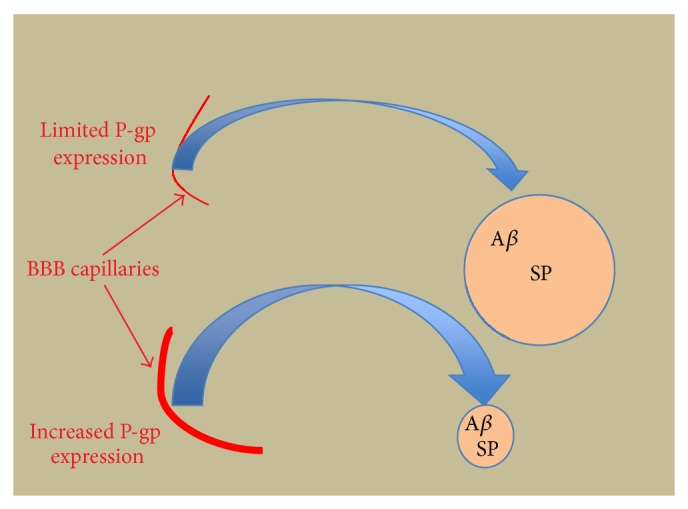
Summative diagram illustrating the putative relationship between capillary P-gp expression, *β*-amyloid burden, and SP development.

Figure 3 summarizes the overall observation that where there were high numbers of SPs, there was a corresponding reduction in the number of P-gp positive capillaries and suggests a mechanistic relationship for this observation based on a P-gp regulatory role in the pathogenesis of *β*-amyloid burden in Alzheimer's disease.

The findings of this study, however, indicate that regional variations in P-gp expression within the brain, and particularly higher levels within the brainstem, may protect against the development of beta amyloid accumulation and senile plaque pathogenesis, and as well, within the superior temporal cortex and perhaps other areas of neocortex, there may be a mechanistic relationship between alterations in P-gp expression as a function of AD disease progression and a progressive accumulation of beta amyloid senile plaque burden. It should be noted that this study did not find any relationship between P-gp expression in the various brain regions examined and neuronal tau levels. As noted previously there is still no established common link between SP and NFT pathogenesis. Insofar as P-gp function is associated with BBB-related transendothelial transport of A*β* from the brain primarily and perhaps exclusively, into the vascular compartment, it should not, perhaps, be surprising that we found no correlation between NFT presence and P-gp capillary expression.

The results of this study lead to further questions such as, for example, within A*β* plaque rich areas of brain such as superior temporal cortex in AD, is there a progressive reduction in P-gp capillary expression as a function of disease progression and time, fundamental to the disease, or is this a secondary alteration contingent upon the development of increased A*β*? In other words, the cause and effect in relationship is not specified by the results of this study, although a causal mechanistic relationship is implied and supported by these observations.

There are biomarker techniques being evaluated to determine A*β* and tau activities as possible diagnostic tools [36]. A diminished P-gp expression that results in SP pathogenesis could perhaps be remediated by introducing P-gp into the brain vasculature or by triggering its increased production. Studies that examine P-gp imaging and measurement techniques in human brains [35, 37] have demonstrated the potential for developing diagnostic tools. In addition, there are currently studies underway to identify pharmaceutical agents which could address the possibility of using P-gp to help reduce or eliminate A*β* from the brain [30, 38, 39]. This, in concert with other approaches [40], could ultimately lead to significant amelioration of the pathogenesis of SP lesion burden.

## 5. Conclusion

Overall, the results of this study suggest an increased protective role against A*β* peptide accumulation and senile plaque pathogenesis in the brainstem (at least within the pons) as a result of increased site specific (pontine) blood-brain barrier P-gp expression. Furthermore, a potential mechanistic relationship between alteration and activity of P-gp capillary expression and A*β* senile plaque development is suggested by the significant inverse correlation noted within superior temporal cortex, a known A*β* and senile plaque rich area of Alzheimer's disease brain. This mechanistic hypothesis suggests that in general, impairing P-gp function leads to increased A*β* load within the brain and senile plaque development. The overall dynamics of A*β* peptide transport and handling between the neural and nonneural compartments across the blood-brain barrier require further and more detailed analysis integrating the P-gp efflux transporter function with the activities of other key transport proteins such as LRP and RAGE. Furthermore, P-gp and the ability to modulate its activity may be an attractive therapeutic target to be considered in future studies.

## Figures and Tables

**Table 1:** Age, gender, and neuropathologic descriptions for each case in the normative and Alzheimer conditions.

Case	Normative				Alzheimer			
	Gender	Age	Braak stage	Cerad level	Gender	Age	Braak stage	Cerad level
1	M	63	0	N/A	F	75	6	High
2	F	73	0	N/A	F	74	6	High
3	M	68	0	N/A	F	84	5/6	High
4	M	81	0	N/A	M	83	1/2	High
5	F	70	0	N/A	F	81	5/6	High
6	F	63	0	N/A	F	81	3/4	High
7	F	65	0	N/A	F	83	5/6	High
8	F	59	0	N/A	M	84	3/4	Mod.
9	M	75	0	N/A	M	76	3	High
10	M	71	0	N/A	F	84	3/4	Mod.
11	F	57	0	N/A	M	74	3/4	High
12	F	71	0	N/A	F	77	5	High
13	M	75	0	N/A	M	60	5/6	High
14	M	74	0	N/A	M	82	3	Mod.
15	F	64	0	N/A	M	88	6	High

**Table 2:** Raw count data for the presence of SPs and P-gp positive capillaries in each condition and site.

Condition	Site	SP_40_	SP_42_	P-gp capillaries
NM	ST	132	688	1279
	HC	35	42	960
	BS	33	36	1358

AD	ST	986	3776	920
	HC	345	528	1192
	BS	78	83	1482

ST: superior temporal cortex; HC: hippocampus; BS: brainstem.

**Table 3:** Comparison of P-gp data in each region and condition. The values are the highest in the brainstem samples in each condition. In each condition the brainstem values were significantly different than the values for the superior temporal and hippocampus samples.

P-gp expression	ST/HC	BS/HC	ST/BS
NM	ns	*P* < 0.012 (BS > HC)	*P* < 0.002 (BS > ST)
AD	ns	*P* < 0.001 (BS > HC)	*P* < 0.001 (BS > ST)

**Table 4:** Correlation results in the superior temporal cortices between P-gp capillary expression and SP presence.

		SP40	SP42	*r*
NM	ST	—	—	
AD	ST	—	—∗∗	−.299
